# A NAMs-based framework for screening the endocrine-disrupting potential of plastic additives using cross-species molecular docking and *Caenorhabditis elegans*

**DOI:** 10.3389/ftox.2026.1751726

**Published:** 2026-06-09

**Authors:** Chaein Chong, Donghyeon Kim, Keon Kang, Jinhee Choi

**Affiliations:** School of Environmental Engineering, University of Seoul, Seoul, Republic of Korea

**Keywords:** *Caenorhabditis elegans*, chemical screening, endocrine disruptors, molecular docking, new approach methodologies, plastic additives

## Abstract

The increasing global production of plastics has raised concerns about additive chemicals that can leach from polymer matrices and lead to widespread human and environmental exposure. While regulatory attention to endocrine-disrupting chemicals (EDCs) and their association with plastic additives has increased in recent years, hazard information for numerous plastic additives remains limited. Here, we applied an integrated new approach methodologies (NAMs)-based framework combining *in silico* molecular docking and *in vivo Caenorhabditis elegans* assays to efficiently screen the endocrine-disrupting potential of plastic additives. Molecular docking of 162 EU REACH–registered additives was performed against human estrogen and androgen receptors (ERα and AR), followed by cross-species docking using AlphaFold-predicted homology models of the *C. elegans* nuclear receptors (NHR-14 and NHR-69). Chemicals showing strong binding interactions across species were further examined through a systematic literature review focused on ER- and AR-mediated effects. By integrating evidence, 1,3-diphenylpropane-1,3-dione (DBM) and 2-(benzotriazol-2-yl)-4-methylphenol (UV-P) were identified as potential ER and AR disruptors and were subsequently validated through reproductive toxicity assays using *C. elegans* wild-type N2 and loss-of-function mutant strains (*nhr-14* and *nhr-69*). This integrated approach provides a practical NAMs-based prioritization strategy by linking molecular interactions to phenotypic outcomes. By positioning *C. elegans* as a biological bridge between human toxicology and ecotoxicology, the framework supports cross-species hazard characterization within a One Health perspective.

## 1 Introduction

The global production and consumption of plastics have risen at an unprecedented pace over recent decades, resulting in widespread distribution of plastics and associated chemicals in the environment (Gautam et al., 2024). During the production, use, and disposal of plastics, numerous additives can be released and subsequently detected in air, water, soil, and biota (Zeng, 2023). However, the leaching behavior and toxicological profiles of many plastic additives remain poorly characterized, raising concerns about potential human and ecological health risks (Maddela et al., 2023). Of particular concern, accumulating evidence indicates that several classes of plastic additives can interfere with hormonal signaling pathways, demonstrating potential for endocrine-disrupting effects (Darbre, 2020; Ullah et al., 2023).

Regulatory agencies and international organizations have intensified efforts to identify and evaluate endocrine-disrupting chemicals (EDCs). Endocrine disruption has recently been designated as a new hazard class under the revised EU CLP Regulation (Jagadeeswaran and Sriram, 2022). The World Health Organization (WHO) and United Nations Environment Programme (UNEP) have emphasized the need for improved EDC screening (Lamb IV et al., 2014), while European Food Safety Authority (EFSA) and the European Chemicals Agency (ECHA) have issued harmonized guidance for EDC identification under EU legislation (ECHA and EFSA et al., 2018). In addition, the Organization for Economic Co-operation and Development (OECD) has established a conceptual framework that outlines a tiered approach for testing and assessing endocrine activity (OECD, 2018). A central principle emphasized across these initiatives is that determination of endocrine-disrupting potential requires not only identification of early molecular perturbations, such as receptor binding, but also demonstration of biologically plausible links to adverse phenotypic effects.

The adverse outcome pathway (AOP) framework provides a mechanistic basis for toxicity assessment by connecting molecular initiating events (MIEs) to organism-level adverse outcomes (AOs) (Ankley et al., 2010). In this context, *in silico* molecular docking can screen chemicals at the MIE level in AOPs, offering a rapid and cost-efficient method for predicting receptor-ligand interactions (Kolšek et al., 2014; Kenda and Sollner Dolenc, 2020). In our previous study (Kim et al., manuscript in preparation), we developed a molecular docking-based prioritization approach and applied it to screen plastic additive chemicals. However, binding scores predicted by *in silico* molecular docking have inherent limitations in accurately assessing the true binding activity of chemicals. In addition, the MIEs identified through molecular docking alone cannot guarantee a linkage to AOs, necessitating experimental validation approaches that connect molecular interactions to organism-level phenotypes.

To address this gap, *C. elegans* has emerged as a valuable non-vertebrate model for linking MIEs to AOs. Approximately 60%–80% of human genes have identifiable orthologs in *C. elegans*, including members of the nuclear hormone receptor superfamily (Lai et al., 2000; Kaletta and Hengartner, 2006; Shaye and Greenwald, 2011). Its short life cycle, genetic tractability, and capacity to measure diverse endpoints make it highly suitable for phenotypic screening.

Building on these findings, the present study aimed to apply a combined approach using molecular docking and *C. elegans* to screen plastic additive chemicals, with a particular focus on endocrine-disrupting effects. This approach demonstrates how NAMs can be effectively applied within the AOP framework and strengthens chemical screening and prioritization strategies aligned with the OECD Conceptual Framework for endocrine disruptor assessment.

## 2 Materials and methods

### 2.1 Study design

**FIGURE 1:**
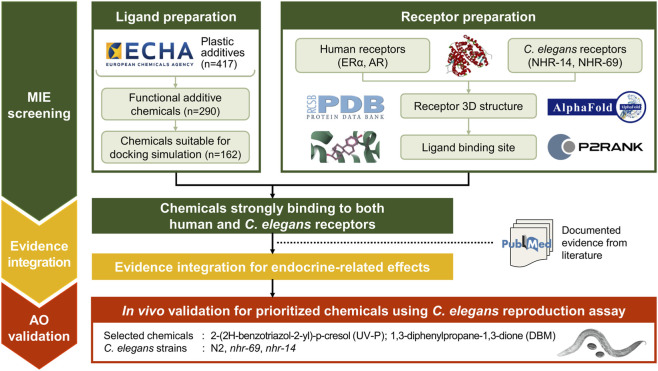
Study workflow.

This study developed and applied an integrated *in silico*-*in vivo* framework for screening the endocrine-disrupting potential of plastic additives (Figure 1). The workflow progressed sequentially through MIE screening, evidence integration, and AO validation.

For MIE screening, *in silico* molecular docking was performed to identify chemicals with potential receptor-binding activity. The chemical library comprised plastic additives from ECHA’s Plastic Additives Initiative (n = 417), refined to structurally suitable compounds (n = 162) for docking simulation. Receptor targets included human ERα and AR alongside their *C. elegans* orthologs NHR-14 and NHR-69, enabling cross-species comparison of binding potential. Human receptor structures were obtained from the Protein Data Bank (PDB) with ligand binding sites defined from crystal structures, whereas *C. elegans* receptor structures were predicted using AlphaFold with binding sites predicted using P2Rank. Molecular docking simulations were performed using AutoDock Vina.

The evidence integration step involved identifying existing data on endocrine-disrupting effects. Chemicals showing strong binding to both human and *C. elegans* receptors were prioritized for literature review. Integrating docking results with reported biological effects allowed prioritization of chemicals most likely to exhibit receptor-mediated activity.

For AO validation, *C. elegans* was used as an alternative *in vivo* model. Reproductive toxicity was selected as the apical endpoint, reflecting a conserved outcome downstream of ER/AR perturbation. Reproduction assays were performed in wild-type N2 and in loss-of-function mutants (*nhr-14* and *nhr-69*) to assess receptor-specific contributions.

### 2.2 Preparation of ligands

#### 2.2.1 Plastic additive chemical dataset

Plastic additive chemicals were sourced from the Plastic Additives Initiative Mapping Exercise, a joint project by the European Chemicals Agency (ECHA) and industrial stakeholders (https://echa.europa.eu/mapping-exercise-plastic-additives-initiative). This initiative identified 417 functional additives used in plastics, focusing on substances registered under EU REACH regulation at quantities exceeding 100 tonnes per year. The dataset categorizes substances by function, including plasticizers, flame retardants, antioxidants, antistatic agents, nucleating agents, stabilizers, and pigments. We initially selected 290 functional additives, excluding pigments due to their insolubility and limited potential for migration.

#### 2.2.2 Ligand structure preparation

Three-dimensional molecular structures were retrieved from PubChem (https://pubchem.ncbi.nlm.nih.gov/) in SDF format using CAS registry numbers. Retrieved SDF files were processed using RDKit to generate optimized 3D conformers with a maximum of 200 embedding attempts.

Compounds unsuitable for molecular docking were excluded, including inorganic substances (e.g., metals), macromolecules (e.g., polymers), complex formulations (e.g., mixtures, reaction masses, nanomaterials), and substances lacking retrievable 3D structural information. Information on the initial 417 substances, including compound-specific exclusion rationales, is summarized in Supplementary Table S1.

### 2.3 Preparation of human and *Caenorhabditis elegans* receptors

Crystal structures of human estrogen and androgen receptors (ERα and AR) were obtained from the RCSB Protein Data Bank (https://www.rcsb.org/). Both agonist and antagonist-bound conformations were selected: ERα with 17β-estradiol (PDB: 1GWR, 2.40 Å) and raloxifene (PDB: 7KBS, 1.83 Å); AR with testosterone (PDB: 2AM9, 1.64 Å) and bicalutamide (PDB: 1Z95, 1.80 Å). Co-crystallized ligands, water molecules, and non-standard residues were removed prior to preparation. Hydrogen atoms were added and protonation states were assigned at pH 7.4 using PDB2PQR with PROPKA. Gasteiger charges were assigned, and structures were converted to PDBQT format.

*Caenorhabditis elegans* NHR-14 (UniProt: O02151) and NHR-69 (UniProt: P91829) were selected as functional orthologs of human ERα and AR based on sequence similarity and experimentally validated functional conservation**.** NHR-14 exhibits estrogen-binding activity and regulates vitellogenin expression (Mimoto et al., 2007), while NHR-69 mediates testosterone-induced reproductive responses (Gamez-Del-Estal et al., 2014; Meng et al., 2024).

Full-length predicted structures were retrieved from the AlphaFold Protein Structure Database (https://alphafold.ebi.ac.uk/). Ligand-binding domain (LBD) regions were cropped based on UniProt domain annotations (NHR-14: residues 131–355; NHR-69: residues 93–344), extended by ±10 residues, and processed identically to human receptors. AlphaFold-predicted structures were subjected to backbone-restrained energy minimization. Binding sites were predicted using P2Rank v2.5 (https://prankweb.cz/) with AlphaFold-specific configuration, incorporating predicted Local Distance Difference Test (pLDDT) scores as confidence features. Structural quality was assessed using PROCHECK Ramachandran analysis and SWISS-MODEL QMEANDisCo scores.

### 2.4 Molecular docking simulations

The method used in this study for molecular docking simulation was adopted from our previous study (Kim et al., manuscript in preparation). Molecular docking was performed using AutoDock Vina (version 1.2.7) with an exhaustiveness parameter of 25. Search space was defined using 30 × 30 × 30 Å cubic grid boxes centered on the LBD. For human receptors, grid coordinates were determined based on crystallographic binding sites. For *C. elegans* receptors, grid boxes were positioned at the highest-scoring P2Rank-predicted sites (Supplementary Table S2). Binding affinity scores (kcal/mol) and a simplified root-mean-square deviation (RMSD)-based pose clustering metric (number of poses with RMSD <2.0 Å) were used to evaluate docking results.

### 2.5 Docking protocol validation

To validate the docking protocol, redocking studies were performed using co-crystallized ligands extracted from the structures. Redocked poses were compared to their crystallographic conformations by calculating heavy-atom RMSD. Following established validation criteria (Trott and Olson, 2010), RMSD values below 2.0 Å were considered indicative of successful protocol validation, demonstrating that the docking algorithm can accurately reproduce experimentally determined binding modes.

To further evaluate the discriminative capacity of the docking protocol, binding affinity predictions were assessed using a reference set of well-characterized nuclear receptor ligands. This reference set comprised endogenous hormones and representative environmental endocrine disruptors with documented estrogen receptor ERα and AR activities.

### 2.6 Evidence integration for chemical prioritization

To prioritize chemicals for experimental validation, candidates exhibiting strong binding affinities to both human and *C. elegans* receptors were further assessed through integration of regulatory information and published evidence on endocrine-related activities.

Regulatory status was compiled from major chemical management frameworks across multiple jurisdictions, including the European Union (REACH Substances of Very High Concern and ECHA endocrine disruptor assessments), the United States (Toxic Substances Control Act and the Endocrine Disruptor Screening Program), the Republic of Korea (Industrial Safety and Health Act and Chemical Control Act), and Japan (Chemical Substances Control Law).

A structured literature search was conducted in PubMed using the following query: ((Estrogen) OR (Androgen) OR (EDC) OR (endocrine disrupting) OR (endocrine disruptor)) AND ([chemical name] OR [CAS number] OR [common synonyms]). Retrieved articles were screened for relevance based on title and abstract, with studies reporting ER or AR modulation prioritized for detailed review.

Information on chemical use categories was additionally obtained from the PlastChem database (Monclús et al., 2025). Chemicals showing consistent support across data streams were classified as high-priority candidates for AO validation using *C. elegans* reproduction assays.

### 2.7 *Caenorhabditis elegans* reproduction assay

*Caenorhabditis elegans* strains used in this study included wild-type N2, *nhr-14* (tm1473), and *nhr-69* (ok 1926). All strains were obtained from the *Caenorhabditis* Genetics Center (CGC) at the University of Minnesota (Minneapolis, MN, USA). Worms were maintained at 20 °C on nematode growth medium (NGM) agar plates seeded with *Escherichia coli* OP50 according to standard protocols. Age-synchronized L1 larvae were obtained by hypochlorite treatment.

The test chemicals were 1,3-diphenylpropane-1,3-dione (DBM; CAS No. 120-46-7; ≥98% purity) and 2-(benzotriazol-2-yl)-4-methylphenol (UV-P; CAS No. 2440-22-4; ≥97% purity). Bisphenol A (BPA; CAS No. 80-05-7; ≥97% purity), a known estrogen receptor (ER) agonist, and testosterone (T; CAS No. 58-22-0; ≥99% purity), an androgen receptor (AR)-active compound, were included as positive controls. All chemicals were purchased from Sigma-Aldrich (St. Louis, MO, United States).

Stock solutions were prepared in dimethyl sulfoxide (DMSO), and working concentrations were prepared by dilution in complete S-medium (CSM). Vehicle controls received DMSO at concentrations equivalent to the highest test concentration (final DMSO ≤0.5%).

For reproduction assay, synchronized L1 larvae were exposed to test chemicals in CSM supplemented with 0.12% OP50 at 200 worms/mL for 50 h. Individual hermaphrodite worms were then transferred to 96-well plates containing 100 μL CSM with 0.06% OP50 and maintained under identical exposure conditions for an additional 48 h. After 98 h of incubation at 20 °C in the dark, rose bengal (300 mg/L) was added per well and plates were heated to 90 °C for 10 min to stain and immobilize progeny. Wells were spotted onto slides and total eggs and offspring per adult were manually counted under a microscope. Each treatment group included four technical replicates per experiment across three independent biological replicates (n = 3).

### 2.8 *Caenorhabditis elegans* body length measurement

Body length was measured to distinguish receptor-mediated effects from general toxicity. Under the same exposure conditions as the reproduction assay, worms at the L1+98 h time point were stained with rose bengal and heat-killed as described above. Body length was then analyzed using a Leica MZ6 stereomicroscope coupled with LAS image analysis software (v4.12; Leica Microsystems, Wetzlar, Germany). Twenty worms per treatment group were measured.

### 2.9 Statistical analysis

Statistical analyses were performed using R version 4.3.1. Normality and homogeneity of variance were assessed using Shapiro-Wilk and Levene’s tests, respectively. When assumptions were met, one-way ANOVA followed by Tukey’s HSD *post hoc* test was applied. Otherwise, Kruskal–Wallis test followed by Dunn’s *post hoc* test with Bonferroni correction was applied.

## 3 Results

### 3.1 Plastic additive chemical dataset

Of the 417 substances identified in the ECHA Plastic Additives Initiative Mapping Exercise, 162 plastic additives were retained for molecular docking after excluding pigments and compounds unsuitable for docking (Supplementary Table S1). These substances are registered under EU REACH regulation at production volumes exceeding 100 tonnes per year and represent additives intentionally added to plastics to achieve specific physical or chemical effects during processing or in the final material. The dataset comprised plasticizers (28.4%, n = 46), other functions (17.3%, n = 28), antioxidants (14.2%, n = 26), flame retardants (16%, n = 26), light stabilizers (7.4%, n = 12), heat stabilizers (5.6%, n = 11), antistatic agents (3.7%, n = 9), other stabilizers (6.8%, n = 6) and nucleating agents (0.6%, n = 1).

### 3.2 Homology modelling of *Caenorhabditis elegans* receptors

**FIGURE 2:**
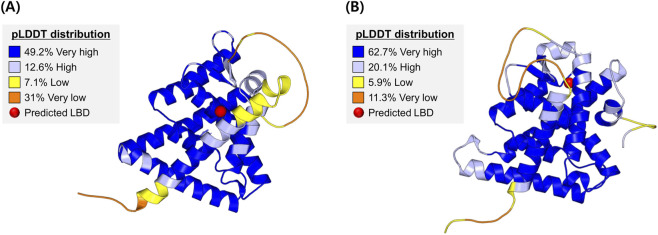
The 3D structure and predicted ligand binding domains of *Caenorhabditis elegans* receptors, **(A)** NHR-14 and **(B)** NHR-69. The protein structures are colored according to AlphaFold confidence scores: blue (very high confidence, pLDDT >90), light blue (high confidence, 70 < pLDDT ≤90), yellow (low confidence, 50 < pLDDT ≤70), and orange (very low confidence, pLDDT <50). Putative ligand binding domains were predicted using P2Rank, and the red spheres indicate the centers of the highest-scoring predicted pockets. Protein structures were visualized using PyMOL.

AlphaFold-predicted structures of NHR-14 and NHR-69 were used in this study (Figure 2). NHR-14 exhibited average pLDDT values of 73.25 for the full-length model and 87.44 for the LBD, while NHR-69 showed a higher average pLDDT of 85.12 for the full-length model and 87.29 for the LBD. Both receptors exceeded the commonly accepted pLDDT threshold of 70 for AlphaFold-based docking studies (Scardino et al., 2023), indicating adequate structural reliability for molecular docking.

Stereochemical quality was assessed by PROCHECK Ramachandran analysis (Laskowski et al., 1993). NHR-14 showed 96.1% of residues in favored and allowed regions with 3.9% in disallowed regions, while NHR-69 achieved 99.7% in favored and allowed regions with no disallowed residues. Global structural quality was evaluated using QMEANDisCo (Studer et al., 2020), yielding scores of 0.50 ± 0.05 for NHR-14 and 0.59 ± 0.05 for NHR-69, comparable to typical values for AlphaFold-predicted structures. Detailed structural quality metrics are summarized in Supplementary Table S3.

Putative ligand-binding sites were predicted using P2Rank. As shown in Figure 2, the top-ranked binding pockets for both receptors were predominantly located within high-confidence regions of the predicted structures, further supporting the suitability of these models for docking-based screening.

### 3.3 Validation of docking protocol

To validate the docking protocol for human receptors, endogenous co-crystallized ligands were re-docked into their cognate receptors. The resulting heavy-atom RMSD values were 1.199 Å for ERα agonist, 0.590 Å for ERα antagonist, 0.455 Å for AR agonist, and 0.979 Å for AR antagonist complexes, all below the commonly accepted threshold of 2.0 Å.

Further validation was conducted using a reference set of well-characterized ligands with established biological activities. The reference panel comprised endogenous hormones, known agonists and antagonists, and compounds reported to exhibit weak or negligible receptor binding.

In accordance with OECD Test Guidelines TG455 and TG458, atrazine and di (2-ethylhexyl) phthalate (DEHP) were included as non-binding reference compounds for estrogen and androgen receptors, respectively (OECD, 2021; 2023). For ERα, diethylstilbestrol (DES) was included as a potent agonist reference (Newbold et al., 2006), while 4-hydroxytamoxifen (4-OHT) and ICI 182,780 (fulvestrant) were used as antagonist controls (Wakeling and Bowler, 1992; Jordan, 2003). Bisphenol A (BPA), reported to act as a weak or partial ER agonist (Delfosse et al., 2012), was included as a low-affinity reference ligand. For AR, 5α-dihydrotestosterone (DHT) and the synthetic androgen R1881 (methyltrienolone) were included as agonist references (Zava et al., 1979), whereas hydroxyflutamide and vinclozolin were used as antagonist references (Simard et al., 1986; Kelce et al., 1994).

Docking results for ERα and AR with their respective reference chemicals are summarized in Supplementary Table S4. Active reference compounds demonstrated strong binding affinities at the key binding sites of their respective receptors, with all active compounds showing binding affinities below −6.0 kcal/mol, while non-binding compounds showed weaker binding.

### 3.4 Molecular docking analysis of plastic additives with human and *Caenorhabditis elegans* receptors

Molecular docking simulations were performed for 162 plastic additives against human receptors (ERα and AR) and their *C. elegans* orthologs (NHR-14 and NHR-69). Binding affinities ranged from −11.2 to −2.8 kcal/mol across all receptors, with median values of −6.3 kcal/mol (ERα), −5.5 kcal/mol (AR), −5.9 kcal/mol (NHR-14), and −5.6 kcal/mol (NHR-69). Complete docking results are provided in Supplementary Table S6.

**FIGURE 3:**
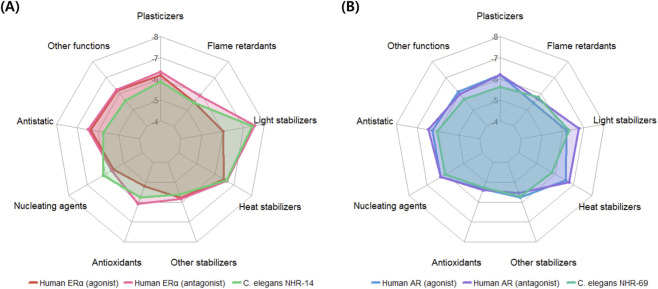
Comparison of binding affinities by plastic additive functional category across human and *Caenorhabditis elegans* receptor orthologs. **(A)** Human ERα and *Caenorhabditis elegans* NHR-14; **(B)** Human AR and *Caenorhabditis elegans* NHR-69. Radar plots show mean binding affinities (kcal/mol) for eight functional use types, revealing binding patterns between human receptors and their *Caenorhabditis elegans* orthologs.

Analysis by functional use type revealed distinct binding patterns across eight plastic additive categories (Figure 3, Supplementary Table S6). Light stabilizers (n = 12) exhibited the strongest binding affinities across all receptors, with mean values ranging from −6.00 to −7.53 kcal/mol depending on the receptor. Heat stabilizers (n = 9) also showed strong binding affinities, with mean values ranging from −5.83 to −6.75 kcal/mol.

To prioritize chemicals for experimental validation, the 10 compounds with the strongest average binding affinities across all six receptor conformations were identified (Table 1). These candidates underwent further evaluation integrating regulatory classifications and literature evidence to guide their prioritization for validation in *C. elegans* reproduction assays.

**TABLE 1:** Top 10 priority plastic additives with strongest predicted receptor binding affinities.

No.	Chemical information			Estrogen receptor Alpha			Androgen receptor		
	Type of function	CAS number	Substance name	ERα agonist (1GWR)	ERα antagonist (7KBS)	NHR-14 (O02151)	AR agonist (2AM9)	AR antagonist (1Z95)	NHR-69 (P91829)
1	Antioxidants	2500-88-1	Di-octadecyl-disulphide	−7.804	−8.784	−7.632	−10.71	−9.31	−7.793
2	Other functions	61788-32-7	Terphenyl, hydrogenated	−8.451	−9.144	−8.261	−8.736	−8.069	−6.697
3	Plasticizers	115-86-6	Triphenyl phosphate	−8.326	−8.85	−7.791	−8.118	−8.551	−6.171
4	Light stabilizers	2440-22-4	2-(2H-benzotriazol-2-yl)-p-cresol (UV-P)	−7.856	−7.682	−8.773	−7.796	−8.338	−7.22
5	Heat stabilizers	101-02-0	Triphenyl phosphite	−8.126	−8.727	−7.625	−8.028	−8.313	−6.299
6	Other stabilizers	120-46-7	1,3-diphenylpropane-1,3-dione	−7.64	−7.137	−7.398	−8.581	−8.129	−8.079
7	Plasticizers	4196-89-8	1,3-Propanediol, 2,2-dimethyl-, 1,3-dibenzoate	−8.524	−7.292	−7.952	−6.679	−8.178	−8.048
8	Light stabilizers	1843-05-6	Octabenzone (UV-531)	−6.697	−8.151	−7.975	−7.573	−7.928	−7.404
9	Plasticizers	27138-31-4	Oxydipropyl dibenzoate	−7.202	−7.147	−7.991	−7.251	−8.064	−7.911
10	Plasticizers	120-55-8	Oxydiethylene dibenzoate (DEGDB)	−7.109	−6.829	−7.595	−8.576	−7.954	−7.475

### 3.5 Evidence-based prioritization of candidate chemicals

Regulatory status of the 10 candidate chemicals revealed that two compounds were already subject to regulatory action. Triphenyl phosphate is listed as a SVHC for endocrine disrupting properties and was designated as an EPA TSCA priority chemical. Terphenyl, hydrogenated was listed as an SVHC for very persistent and very bioaccumulative (vPvB) properties. The remaining eight compounds were not regulated under the assessed frameworks from the EU, United States, Korea, or Japan (Supplementary Table S7).

Systematic literature searches identified 12 relevant publications covering four of the candidate chemicals (Table 2; detailed summaries in Supplementary Table S8). For DBM, the literature indicates a distinct profile of mechanistically supported endocrine-modulating activity, primarily as an ER antagonist. Reported activities include suppression of E2-stimulated proliferation in MCF-7 cells, inhibition of anchorage-independent growth, downregulation of ER-regulated oncogenes, and reduction of ER–ERE binding (Lin et al., 2005; Igarashi et al., 2025). AR-suppressive activity has also been reported, with dose-dependent reductions in AR protein levels, *AR* mRNA expression, and PSA signaling (Jackson et al., 2007).

**TABLE 2:** Summary of endocrine-related evidence for selected low-affinity chemicals identified in docking analysis both in human and *Caenorhabditis elegans*. Regulatory EDC classification reflects current status under EU (REACH SVHC, ECHA ED Assessment), US (EPA TSCA, EDSP), Korean (ISHA, CCA), and Japanese (CSCL) frameworks. Evidence from literature summarizes endocrine-related activities from *in vitro* and *in vivo* studies. Comprehensive regulatory assessment and complete literature citations are provided in Supplementary Tables S5, S6, respectively.

Substance (CAS)	Usetype	Regulatory EDC classification	Evidence from literature (ER/AR)	
			Estrogenic effect	Androgenic effect
1,3-diphenylpropane-1,3-dione DBM (120-46-7)	Heat stabilizer; Light stabilizer; Lubricant	Not classified (ECHA-ED; SVHC)	Suggested (ER antagonism): Antiestrogenic activity observed in subcutaneous uterotrophic assay (Lin et al., 2005; Igarashi et al., 2025)	Suggested (AR expression regulation): Suppression of AR expression reported (Jackson et al., 2007)
2-(2H-benzotriazol-2-yl)-4-methylphenol; UV-P (2440-22-4)	Light stabilizer; Antioxidant; Colorant; Filler; Intermediate	Not classified (ECHA-ED; SVHC)	Suggested (ER agonist): Negative in yeast assays (Morohoshi et al., 2005; Fent et al., 2014), but positive in mammalian cell–based assays (Feng et al., 2020; Sakuragi et al., 2021) suggesting potential relevance in human ER context	Suggested (AR antagonist): Antagonistic activity observed in reporter gene and yeast assays (Fent et al., 2014; Sakuragi et al., 2021)
Octabenzone; UV-531 (1843-05-6)	Light stabilizer; Heat stabilizer; Antioxidant; Colorant; Filler	Not classified (ECHA-ED; SVHC)	Limited: Most studies reported no ER agonist/antagonist activity. Moderate inhibition observed in rat enzymes, but inactive toward human enzymes (Chen et al., 2025)	Limited: No AR agonist/antagonist activity detected in yeast or reporter gene assays (Nashev et al., 2010; Carstensen et al., 2023)
Oxydiethylene dibenzoate; DEGDB (120-55-8)	Plasticizer; Light stabilizer; Biocide; Colorant; Filler; Lubricant	Not classified (ECHA-ED; SVHC)	Limited: Few studies available; existing transactivation assays show no ER agonist/antagonist activity (Lee et al., 2022)	Limited: Few studies available; existing transactivation assays show no AR agonist/antagonist activity (Lee et al., 2022)

UV-P similarly showed strong concordance between *in silico* predictions and published experimental results. Although yeast-based ER assays reported predominantly negative outcomes (Morohoshi et al., 2005; Fent et al., 2014), mammalian cell–based assays consistently demonstrated ERα agonism, including E2-dependent synergistic activation and mechanistic attenuation by ICI 182,780 (Feng et al., 2020). CHO-K1 reporter assays further confirmed ERα/ERβ activation (Sakuragi et al., 2021). For AR, UV-P exhibited no agonist activity but consistently demonstrated antagonistic responses across yeast and mammalian systems (Fent et al., 2014; Sakuragi et al., 2021).

In contrast, octabenzone (UV-531) and oxydiethylene dibenzoate (DEGDB) showed only limited or inconsistent evidence for endocrine activity. For UV-531, most studies reported no measurable ER or AR agonist/antagonist activity (Morohoshi et al., 2005; Webster et al., 2019; Carstensen et al., 2023). Moderate inhibition of rat ovarian 17β-HSD1 was observed (Chen et al., 2025), but the absence of effects in human 17β-HSD1 indicates a species-specific mechanism with unclear human relevance. For DEGDB, only one relevant study was identified (Lee et al., 2022), with both OECD TG 455 ER assays and AR transactivation assays reporting no activity. Due to the lack of mechanistic evidence, both UV-531 and DEGDB were classified as low priority.

Based on this evidence integration, DBM and UV-P were selected as potential endocrine disruptors for experimental validation. Both substances demonstrated concordance between the docking-based receptor binding predictions and the reported ER/AR perturbations.

### 3.6 *In vivo* validation of reproductive toxicity in *Caenorhabditis elegans*

Based on the molecular docking and evidence integration, DBM and UV-P were selected as priority chemicals for experimental validation using *C. elegans* reproduction assays. In preliminary range-finding experiments, DBM at 50 μM caused complete lethality, and UV-P at 500 μM resulted in complete reproductive failure. To examine potential receptor-mediated mechanisms, reproductive outcomes at sub-lethal concentrations were compared among wild-type N2 and the loss-of-function mutants *nhr-14* and *nhr-69*.

**FIGURE 4:**
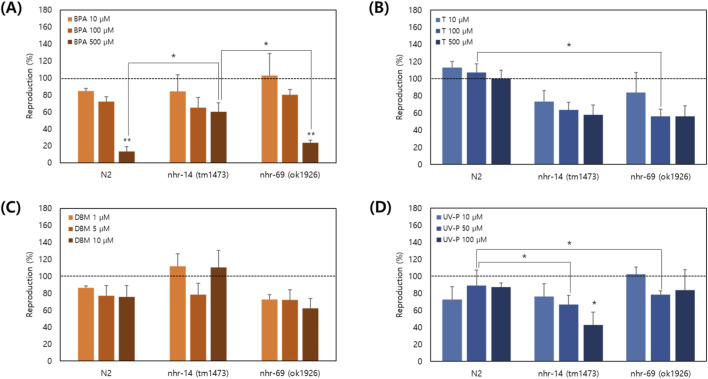
Reproductive outcomes in *Caenorhabditis elegans* strains exposed to test chemicals. Brood size was assessed in wild-type N2, nhr-14 (tm1473), and nhr-69 (ok 1926) mutants following exposure to **(A)** BPA (10–500 μM), **(B)** testosterone (T; 10–500 μM), **(C)** DBM (1–10 μM), and **(D)** UV-P (10–100 μM). Data were normalized to the solvent control (DMSO) and are presented as mean ± SEM (n = 3). Asterisks indicate statistically significant differences compared with the control and among *Caenorhabditis elegans* strains (*p < 0.05, **p < 0.01, ***p < 0.001).

The reference chemicals provided strain-specific response patterns that served as mechanistic anchors. BPA, a known ER agonist, showed partial reproductive recovery in *nhr-14* mutants at 500 μM (Figure 4A), whereas testosterone, an AR-active compound, showed enhanced reproductive toxicity in *nhr-69* mutants (Figure 4B).

DBM exhibited a response pattern comparable to that of BPA. Although the difference did not reach statistical significance, *nhr-14* mutants showed a trend toward higher reproductive output than N2 at 1 and 10 μM (Figure 4C). This recovery was observed despite a significant reduction in body length in *nhr-14* at 10 μM (Supplementary Figure S1C). Given the absence of statistical significance and the complete lethality observed at 50 μM, systemic toxicity rather than NHR-14–mediated signaling likely contributes to the observed response. These findings therefore indicate that the involvement of NHR-14 in DBM-induced reproductive toxicity remains inconclusive.

UV-P showed a response pattern comparable to that of testosterone. Reproductive output was significantly reduced in both *nhr-14* and *nhr-69* mutants relative to N2 at 50 μM, with *nhr-14* showing additional suppression at 100 μM (Figure 4D). Body length in *nhr-69* mutants was not significantly altered at any tested concentration of UV-P (Supplementary Figure S1D), suggesting that the observed reproductive effects were not explained by general cytotoxicity and were consistent with a potential involvement of NHR-69.

## 4 Discussion

### 4.1 Advantages of a molecular docking–*C. elegans* combined approach within the OECD EDC assessment framework

The combined use of molecular docking and *C. elegans* assays offers clear advantages when viewed through the OECD Conceptual Framework (CF) for Testing and Assessment of Endocrine-Disrupting Chemicals. Within this tiered framework, molecular docking corresponds to Level 1, which encompasses existing information and non-testing approaches such as *in silico* predictions. Docking therefore provides an efficient means of identifying potential MIEs, particularly ligand–receptor interactions that may trigger endocrine-disruptive pathways. These mechanistic predictions allow early prioritization of chemicals and support hypothesis generation without requiring vertebrate testing. *Caenorhabditis elegans*, as a whole-organism non-mammalian *in vivo* model, fits within OECD Level 3–4, providing organism-level evidence relevant to endocrine-linked developmental, reproductive, and behavioral endpoints. Because many nuclear receptor pathways and xenobiotic metabolic processes are conserved between *C. elegans* and vertebrates, phenotypic responses in the nematode can offer functional support for docking-derived hypotheses.

Previous work has demonstrated that loss-of-function mutants of nhr-14 and nhr-69 respond reliably to Tox21-active endocrine disruptors, supporting their utility for receptor-targeted screening (Jeong et al., 2019). Building on this foundation, concordance between predicted receptor interactions and observed phenotypes strengthens biological plausibility, whereas discrepancies help distinguish receptor-mediated mechanisms from general toxicity. The integration of Level 1 and Level 3/4 information enhances the overall weight of evidence and aligns with the OECD emphasis on mechanistic understanding, tiered evaluation, and the use of alternative methods prior to vertebrate studies. This multi-level approach further provides a practical framework for chemical prioritization, enabling the early identification of potentially endocrine-active substances and the more efficient allocation of experimental resources.

### 4.2 Regulatory perspective of prioritized plastic additives

Based on the results of this study, UV-P was prioritized as a plastic additive with a high potential for endocrine disruption. UV-P is widely used as a light stabilizer in plastics, coatings, and adhesives, with an estimated global production volume of approximately 20,000 tons annually. Previous studies have reported widespread environmental detection of UV-P in surface water and sediments (Kameda et al., 2011), fish muscle tissue (Kim et al., 2011), plastic products (Sakuragi et al., 2021), and human urine (Mao et al., 2024), indicating actual environmental exposure in the general population.

Despite its extensive use and consistent detection across environmental and biological matrices, UV-P has not been subject to regulatory evaluation for endocrine-disrupting properties, and only a limited number of studies have examined its potential endocrine-related effects. This regulatory gap highlights the need for further research and demonstrates the value of the proposed screening approaches.

### 4.3 Study limitations

Several limitations should be recognized when interpreting the findings of this study. First, the use of AlphaFold-predicted structures as docking targets for the *C. elegans* nuclear receptors NHR-14 and NHR-69 introduces inherent uncertainty. AlphaFold models do not account for ligand-induced conformational changes, which are particularly important for nuclear receptors that undergo substantial structural rearrangements upon ligand binding. As computationally predicted structures rather than experimentally resolved crystal conformations, the reliability of binding pocket geometry remains limited. While structural quality was evaluated using PROCHECK Ramachandran plot analysis and QMEAN scoring, these metrics cannot fully resolve potential uncertainty. Therefore, docking results for *C. elegans* receptors should be interpreted as comparative, screening-level estimates intended for relative ligand prioritization rather than definitive measurements of binding affinity.

Second, species differences between *C. elegans* and humans must be acknowledged. Receptor selection was based on published evidence of sequence similarity and partial functional conservation with human receptors. Owing to evolutionary divergence between nematodes and mammals, however, NHR-14 and NHR-69 cannot be regarded as complete functional equivalents of human ER and AR. Residue-level differences in the ligand-binding pocket between orthologous nuclear receptors can alter the functional outcome of ligand engagement, such that the same compound may act as an agonist in one species while functioning as an antagonist in another (Bridgham et al., 2006). In the present study, UV-P elicited a reproductive phenotype comparable to that of testosterone in *C. elegans*, despite literature evidence indicating predominant AR antagonism in mammalian *in vitro* systems. Such discrepancies likely reflect architectural divergence between nematode NHRs and mammalian receptors rather than inconsistency in the underlying chemical activity.

A related challenge arises from the hermaphroditic nature of *C. elegans* which complicates the mechanistic separation of ER- and AR-related effects. *Caenorhabditis elegans* hermaphrodites undergo spermatogenesis during the L4 larval stage followed by a transition to oogenesis in adulthood, resulting in the coexistence of male and female reproductive processes within a single organism (Nishimura and L’Hernault, 2010). Given this inherent difficulty in separating ER- and AR-specific responses in hermaphrodites, chemicals predicted to bind to both receptor types were prioritized for experimental validation in order to capture overall endocrine-disrupting activity at the organism level rather than to resolve individual receptor pathways.

Taken together, these limitations suggest that the present findings should be interpreted as screening-level evidence for chemical prioritization rather than definitive mechanistic confirmation. Nevertheless, the integrated docking and *C. elegans* approach provides a practical framework for prioritizing plastic additives with potential endocrine-disrupting activity and for guiding further mechanistic and regulatory evaluation.

## 5 Conclusion

This study developed an integrated NAMs-based screening framework combining *in silico* cross-species molecular docking with *in vivo C. elegans* assays to prioritize plastic additive chemicals. This framework provides a scalable, mechanistically informed strategy for chemical prioritization that aligns with the OECD Conceptual Framework for EDC assessment and the 3 R s principles. Furthermore, by employing *C. elegans* as a bridging model encompassing both human and ecological receptor systems, the approach advances cross-species hazard characterization within a One Health perspective.

## Data Availability

The original contributions presented in the study are included in the article/Supplementary Material, further inquiries can be directed to the corresponding author.
