# Supplementary material for: A NAMs-based framework for screening the endocrine-disrupting potential of plastic additives using cross-species molecular docking and Caenorhabditis elegans
Source: Front Toxicol. 2026 Jun 9;8:1751726. doi: 10.3389/ftox.2026.1751726 (PMC13286444; doi:10.3389/ftox.2026.1751726)
Supplement: Supplementary file 1 [file Image1.pdf]

# *Supplementary Material*

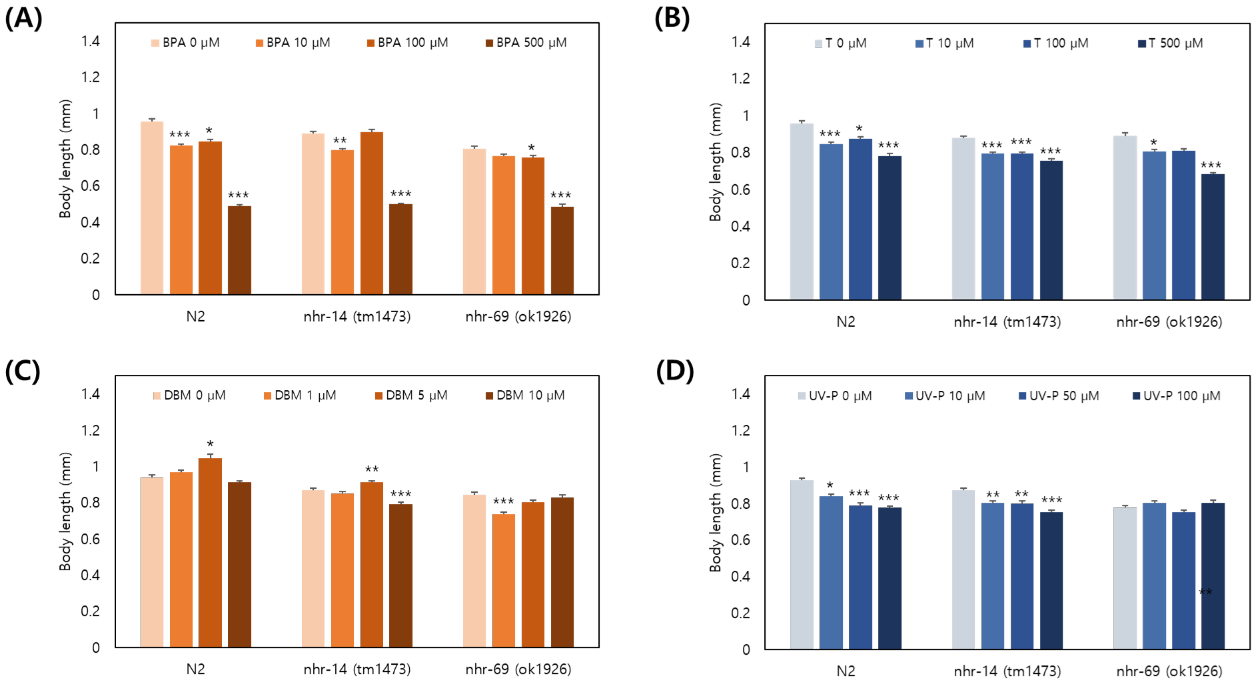


**Supplementary Figure 1. Body length in *C. elegans* strains exposed to test chemicals.** Body length was assessed in wild-type N2, *nhr-14* (tm1473), and *nhr-69* (ok1926) mutants following exposure to (A) BPA (10–500 μM), (B) testosterone (T; 10–500 μM), (C) DBM (1–10 μM), and (D) UV-P (10–100 μM). Data are presented as mean ± SEM (n = 20). Asterisks indicate statistically significant differences compared with the solvent control (DMSO) (* $p < 0.05$, ** $p < 0.01$, *** $p < 0.001$).
